# *Bacillus pumilus *laccase: a heat stable enzyme with a wide substrate spectrum

**DOI:** 10.1186/1472-6750-11-9

**Published:** 2011-01-25

**Authors:** Renate Reiss, Julian Ihssen, Linda Thöny-Meyer

**Affiliations:** 1 Empa, Swiss Federal Laboratories for Materials Science and Technology, Laboratory for Biomaterials, Lerchenfeldstr.5, 9014 St. Gallen, Switzerland

## Abstract

**Background:**

Laccases are multi-copper oxidases that catalyze the one electron oxidation of a broad range of compounds. Laccase substrates include substituted phenols, arylamines and aromatic thiols. Such compounds are activated by the enzyme to the corresponding radicals. Owing to their broad substrate range laccases are considered to be versatile biocatalysts which are capable of oxidizing natural and non-natural industrial compounds, with water as sole by-product.

**Results:**

A novel CotA-type laccase from *Bacillus pumilus *was cloned, expressed and purified and its biochemical characteristics are presented here. The molecular weight of the purified laccase was estimated to be 58 kDa and the enzyme was found to be associated with four copper atoms. Its catalytic activity towards 2,2'-azino-bis(3-ethylbenzthiazoline-6-sulphonic acid) (ABTS), 2,6-dimethoxyphenol (2,6-DMP) and syringaldazine (SGZ) was investigated. The kinetic parameters *K*_M _and *k*_cat _for ABTS were 80 ± 4 μM and 291 ± 2.7 s^-1^, for 2,6-DMP 680 ± 27 μM and 11 ± 0.1 s^-1 ^and for SGZ only *k*_cat _could be estimated to be 66 ± 1.5 s^-1^. The pH optimum for ABTS was 4, for 2,6-DMP 7 and for SGZ 6.5 and temperature optima for ABTS and 2,6-DMP were found to be around 70°C. The screening of 37 natural and non-natural compounds as substrates for *B. pumilus *laccase revealed 18 suitable compounds. Three of them served as redox mediators in the laccase-catalyzed decolorization of the dye indigocarmine (IC), thus assessing the new enzyme's biotechnological potential.

**Conclusions:**

The fully copper loaded, thermostable CotA laccase from *Bacillus pumilus *is a versatile laccase with potential applications as an industrial biocatalyst.

## Background

Laccases (EC 1.10.3.2), together with ferroxidases (EC 1.16.3.1), ascorbate oxidase (EC 1.10.3.3) and ceruloplasmin (EC 1.16.3.1) belong to the multi-copper oxidase (MCO) enzyme family. These enzymes are classified as blue copper proteins and contain between one and six copper atoms. They are produced by various fungi, plants, insects and bacteria [1]. They catalyze the oxidation of a range of organic substances *via *a four-electron reduction of oxygen to water [2,3].

Laccase catalyzed reactions include the polymerization of monomers, degradation of polymers and the oxidation of phenolic compounds [4]. Laccases can act on non-phenolic compounds by employing mediators, which undergo an oxidation-reduction cycle, thus shuttling electrons between the non-phenolic compound and the enzyme. These laccase mediator systems (LMS) have been used in a number of processes including pulp delignification and oxidation of organic pollutants [5].

Besides being commercially used in denim bleaching, laccases have also been applied in the decolorization and transformation of textile dyes. Dye effluents from the textile industry represent a major environmental pollutant, and conventional degradation processes suffer from inefficiency, are not price competitive or result in toxic by-products [6-9].

Numerous studies have shown that laccase catalyzed decolorization of textile dyes was achieved either by direct oxidation or *via *indirect oxidation using mediators, which enhanced or enabled the reaction [7,10]. Preferably, mediators should be good laccase substrates, stable in oxidized and reduced form and have no inhibitory effect upon enzyme activity. Once oxidized, mediators interact between the active site of the enzyme and the target molecule, thus broadening the range of substrates which can be oxidized. Well established non-natural laccase mediators are ABTS, TEMPO (2,2,6,6-tetramethyl-piperidine-1-oxyl radical) and HBT (1-hydroxybenzotriazole). However, the emphasis on natural mediators has gained momentum due to economic and ecological awareness [11,12].

To date, only fungal laccases are industrially relevant for the detoxification of synthetic dyes and other applications owing to their higher redox potential compared to bacterial laccases [4,5,13-16]. However, more recently bacterial laccases have also been shown to successfully oxidize dyes in the presence and absence of redox mediators [17-19]. Developing bacterial laccases for biotechnological applications will be advantageous because they are sustainable and can be produced in a short time in inexpensive media. Fungal laccases suffer from several drawbacks, such as lack of functional or efficient expression in heterologous hosts, relatively long fermentation times and comparatively low yield. Furthermore, as bacterial laccases are suitable for heterologous expression in *E. coli*, the enzyme can easily be tailored using techniques such as directed evolution [20]. Based on the intrinsic properties of bacterial laccases, these enzymes have great potential as biocatalysts for oxidation reactions, as they operate in aqueous solvent at neutral to basic pH, are temperature stable, cofactor-independent and produce water as a sole by-product [21].

The endospore coat protein CotA from *Bacillus subtilis *is a well-studied thermostable bacterial laccase which has been crystallized. It is believed to play a role in the biosynthesis of the brown spore pigment, which might be involved in the UV light protection of the spore coat [22]. CotA contains a four-copper active site with the type 1 blue copper centre T1 and the T2/T3 trinuclear cluster (one type 2 and two type 3 copper atoms) [22,23]. The one electron oxidation of the substrate to the corresponding radical takes place at the T1 site and the abstracted electron is transferred to the trinuclear centre, where dioxygen is reduced to water [24]. Other bacterial laccases have been described in *Azospirillum lipoferum*, *Escherichia coli*, *Bacillus licheniformis*, *Bacillus halodurans*, *Streptomyces coelicolor*, *Thermus thermophilus *and *γ-Proteobacterium JB*, to mention the most relevant examples [4,19,25]. Bacterial laccases are characterized by their high temperature tolerance and high levels of activity in neutral to alkaline conditions, whereas fungal laccases usually operate under acidic pH.

We have identified a CotA-type laccase from *Bacillus pumilus *by genome mining which differed by over 30% in its amino acid sequence from the *B. subtilis *laccase. We cloned the corresponding gene, purified the protein and characterized it biochemically. The purified laccase was tested for its ability to decolorize indigocarmine (IC), a compound belonging to the group of carbonyl dyes which are widely used in the industrial production of denim. The decolorization of this dye remains challenging and enzymatic decolorization using the laccase directly or as LMS has great potential.

## Methods

### Materials

2,2'-Azino-bis(3-ethylbenzthiazoline-6-sulphonic acid) (ABTS), 4-hydroxy-3,5-dimethoxybenzaldehyde azine (syringaldazine, SGZ), 2,6-dimethoxyphenol (2,6-DMP), 3', 5'-dimethoxy-4'-hydroxyacetophenone (acetosyringone (ACS)) and indigocarmine (IC) were purchased from Sigma-Aldrich. All other chemicals were standard reagent grade (Sigma Aldrich).

### Bacterial strains and plasmids

*B. pumilus *DSM 27 was obtained from the German collection of microorganisms (DSMZ). *E. coli *strain JM109 [genotype *endA*1 *recA*1, *gyrA*96, *thi*, *hsdR*17,(r_K_^-^, m_K_^+^), *relA*1, *supE*44, λ^-^, Δ*(lac-proAB)*, (F', *traD*36, *proAB*, *lacI*^q^ZΔM15)] and the pQE-60 expression vector were purchased from Promega (Madison, USA) and Qiagen (Valencia, USA), respectively. The origin of replication in pQE-60 is ColE1 (pBR322) and transcription of the inserted gene is controlled by the bacteriophage T5 promoter (recognized by the *E. coli *housekeeping RNA polymerase) and two *lac *operator sequences (conferring inducibility by IPTG). For efficient repression the host strain JM109 which over-expresses the repressor LacI was used.

*B. pumilus *was grown overnight at 30°C and 150 rpm in SRB medium (20 g/L yeast extract

25 g/L tryptone, 3 g/L K_2_HPO_4_). *E. coli *was routinely grown in LB medium at 37°C and 150 rpm. For plasmid selection 0.1 mg/mL ampicillin was added to LB agar plates and liquid medium.

### Cloning of the *B. pumilus *laccase gene

The putative laccase gene (spore coat protein A, *cotA*) of *B. pumilus *was identified by Protein Blast (http://blast.ncbi.nlm.nih.gov/Blast.cgi) using the CotA sequence of *B. subtilis *as search template (gene bank accession no. NP_388511). The *cotA *gene of *B. pumilus *(accession no. ZP_03054403) was PCR amplified from genomic DNA with forward primer 5'- CGT CC ATG GAA AAC CTA GAA AAA TTT GTT GAC GAG C -3' (introducing an *Nco*I site around the native start codon and an additional glutamate codon after ATG) and reverse primer 5'- CCATG AAGCTT TTA CTG GAT GAT ATC CAT CGG C-3' (introducing a *Hind*III site after the native stop codon). PCR was performed with high fidelity Phusion polymerase (New England Biolabs, Ipswich, USA) and appropriately diluted cell suspension of *B. pumilus *DSM 27 as template. The 1552 bp PCR product was cloned into the high copy number cloning vector pJet/Blunt (Fermentas, St. Leon-Rot, Germany) by blunt end ligation. After sequence verification the 1542 bp *Nco*I-*cotA*-*Hind*III fragment of pJet/Blunt-*cotA *was sub-cloned into the *Nco*I and *Hind*III sites present in the multiple cloning site of pQE-60, resulting in plasmid pBpL2. The *NcoI *site of pQE-60 contains the start codon for expression of cloned genes. In order to obtain fully wild-type CotA of *B. pumilus *the additional glutamate codon introduced at position 2 for cloning purposes was removed from pBpL2 by quick change mutagenesis using forward primer 5'-GAGGAGAAATTAACC **ATG AAC **CTA GAA AAA TTT GTT GAC-3' and complementary reverse primer 5'-GTC AAC AAA TTT TTC TAG **GTT CAT **GGTTAATTTCTCCTC-3'. A map of the final expression plasmid pBpL6 containing the *cotA *gene as confirmed by DNA sequencing is shown in the Additional file 1.

### CotA production

*E. coli *JM109 was transformed with pBpL6 for expression and purification of *B. pumilus *laccase.

To obtain a fully copper loaded enzyme, the method reported by Durao *et al*. 2008 was used for CotA production [26]. Briefly, the recombinant *E. coli *strain was cultivated in TB medium supplemented with 0.1 mg/mL ampicillin at 30°C and 120 rpm. Starting from an isolated colony, an overnight pre-culture was diluted 1:50 into a 700 mL volume in a 2 L Erlenmeyer flask. At an optical density of 1-2 (ΔOD_600_), laccase expression was induced by adding 0.1 mM IPTG. At the same time, 0.25 mM CuCl_2 _was added and the temperature was decreased to 25°C. The cells were further incubated for 4 h at 120 rpm and then without shaking for additional 16 hours. Durao *et al*. (2008) showed that static incubation improved the cellular copper content, thus yielding a fully copper loaded protein population. Cells were harvested by centrifugation at 4°C for 30 min at 4,495 × *g*, washed in 20 mM Tris buffer pH 7.6, centrifuged again and subsequently stored at -20°C.

Frozen cells were thawed on ice and resuspended in 20 mM Tris buffer pH 7.6 containing 1 mg/mL lysozyme and protease inhibitor mix (Roche Complete Protease Inhibitior Mix, EDTA-free) and re-frozen at -80°C. Cells were thawed, Benzonase^® ^Nuclease (Roche) was added and the suspension incubated for 1 h at 37°C at 120 rpm. The suspension was subjected to twelve 10 s rounds of sonication with a Branson sonicator equipped with a microtip at a setting of 80%. Cellular debris was removed by centrifugation at 4°C for 40 min, 47,000 × *g*. The crude cell lysate was incubated at 70°C for 20 min and soluble protein was collected by centrifugation at 4°C for 40 min and 47,000 × *g*. Purification was performed on an Äkta purifier FPLC system (GE-Healthcare). The sample was loaded onto a 27 mL Q-Sepharose FF anion exchange chromatography column (GE-Healthcare), previously equilibrated with 20 mM Tris buffer pH 7.6. Proteins were eluted with a NaCl gradient from 0 to 1 M. Fractions displaying laccase activity, as detected using an ABTS oxidation assay, were pooled and concentrated by ultrafiltration using a 30 kDa cut-off. The sample was loaded onto a Superdex 75 column (GE-Healthcare), previously equilibrated with 20 mM Tris buffer containing 0.1 M NaCl pH 7.6. Fractions with laccase activity were pooled, concentrated by ultrafiltration and stored in 20 mM Tris buffer pH 7.6 at -80°C. The purity of the sample was analyzed by SDS-PAGE using a 10% polyacrylamide gel. The copper content was determined by ICP-OES (Inductive coupled plasma optical emission spectroscopy) on a Perkin Elmer Optima 3000 instrument. The protein solution was treated with nitric acid in a microwave digestion system prior to optical analysis at Cu lines 327.393 nm and 324.752 nm.

### Determination of protein and enzyme activities

Total protein concentration was determined by the method of Bradford [27] with bovine serum albumin (BSA) as standard and by UV-Vis absorbance measurements with a Nanodrop ND-1000 spectrophotometer using an extinction coefficient of 77,303 M^-1^cm^-1 ^(calculated from the amino acid sequence).

Spectrophotometric laccase activity assays were routinely carried out in a 96-well plate at 25°C with 0.5 mM ABTS in McIlvaine buffer at pH 4 (mixture of 0.1 M citric acid and 0.2 M K_2_HPO_4_) using a Varian Cary 50 Bio or a Bio Tek Synergy Mx spectrophotometer and initiated by adding enzyme solution. The assay volume was either 200 or 300 μL.

Oxidation of ABTS was monitored at 420 nm (ε = 36,000 M^-1^cm^-1^), of SGZ at 525 nm (ε = 65,000 M^-1^cm^-1^) and of 2,6-DMP at 468 nm (ε = 37,500 M^-1^cm^-1^). Laccase activity as a function of pH was performed in McIlvaine buffer in the pH range 2-8 for ABTS (0.5 mM), SGZ (0.05 mM) and 2,6-DMP (0.3 mM). The temperature optimum was recorded between 20°C and 75°C by following ABTS or 2,6-DMP oxidation in an assay volume of 3 mL using a magnetically stirred, temperature controlled cuvette device. The effect of DMSO on laccase activity was determined by adding 0.4-50% (v/v) of solvent to the reaction mixture and following the oxidation of ABTS, SGZ or 2,6-DMP. The stability of laccase was tested by incubating the enzyme in McIlvaine buffer at pH 5 or 7, potassium phosphate buffer pH 7 and in ddH_2_O at 4, 25, 45 and 65°C and subsequently performing an ABTS assay.

Kinetic parameters of purified laccase were determined in McIlvaine buffer at 25°C against ABTS (10 mM-0.6 μM, pH 4), 2,6-DMP (10 mM-0.6 μM, pH 7) and SGZ (1.25 mM-0.04 μM plus 25% (v/v) DMSO, pH 6.5). Enzymatic assays were performed in triplicate. Oxidation of ACS was monitored at 340 nm by the decrease of substrate absorbance (ε = 1,000 M^-1^cm^-1^) in 0.1 M potassium phosphate buffer (2.5 mM-0.04 mM, pH 6.5). The initial rates, recorded within 3 to 10 min, were approximated by non-linear regression algorithms according to the Michaelis-Menten equation using SIGMA-PLOT Enzyme Kinetics software. One unit was defined as the amount of enzyme that oxidized 1 μmol of substrate per minute.

### Substrate/mediator screening and IC decolorization

Purified laccase was subjected to a substrate screening. The screening was based on the direct oxidation of the substrate and on the coupled decolorization of IC. Therefore, potential laccase substrates were dissolved in water and 5% (v/v) DMSO at a concentration of 10 mM and diluted to a final concentration of 1 mM. Reactions were performed in 96-well plates in 0.1 M potassium phosphate buffer, pH 6.5 or 7.8 at 37°C with shaking. The reaction was initiated by adding 0.23 U/mL purified laccase. One unit was defined as the amount of enzyme that oxidized 1 μmol of ABTS per minute at room temperature. A UV-Vis scan between 230-700 nm was recorded prior to laccase addition and after 48 hours reaction time.

To evaluate the ability of the purified laccase to decolorize IC, 1 mM of the dye was dissolved in water and incubated with 0.1 mM of the potential mediator substrate. The reaction was initiated by the addition of laccase which was substituted with water in the control reaction. The reaction was followed over a period of 48 h at intervals of 30 min and was performed at 37°C. The change in absorbance (for λ = 350, 420, 550, 570, 610 and 650 nm) was recorded to follow IC decolorization. Although IC showed a maximum absorbance at 610 nm, decolorization was calculated based on the shoulder at 650 nm. In this way it was possible to follow starting concentrations of 1 mM as the Lambert-Beer law was still valid under these conditions. Furthermore, no by-product was detected at 650 nm as opposed to 610 nm. The decolorization was further assessed by varying the IC to ACS ratio in the presence and absence of laccase.

Decolorization (%) was calculated based on the difference in absorbance at 650 nm in reactions with and without laccase and defined as:

$$\text{Decolorization}\left(\%\right)=100-\frac{absorbance_{t48}{\_}_{laccase}\times 100}{absorbance_{t48\_control}}$$

Decolorization reactions were carried out in the presence and in the absence of laccase. The absorbance measured after 48 h in reactions lacking laccase was then defined as 100% (absorbance_t48_control_).

## Results

### Cloning of *cotA *from *Bacillus pumilus*

A Protein Blast search for uncharacterized bacterial laccases was performed using the CotA amino acid sequence of *Bacillus subtilis *as template. Out of numerous homologues, a hypothetical protein annotated as "spore coat protein A" found in the fully sequenced genome of *Bacillus pumilus *ATCC 7061 was selected for cloning and recombinant expression in *E. coli *due to its relatively close relation to other already characterized *Bacillus *laccases. The *B. pumilus *DSM 27 sequence encoding a CotA-like protein was cloned into the expression vector pQE-60 as described in Methods. The cloned sequence exhibited 99.8% amino acid identity to the CotA sequence of *B. pumilus *ATCC7061, with K79E being the only difference. CotA of *B. pumilus *DSM 27 exhibited 68% amino acid identity and 79% similarity to CotA of *B. subtilis*, which is the best studied bacterial laccase [22,28]. Amino acid identity and similarity to the second previously characterized CotA laccase from *B. licheniformis *ATCC 7061 (accession no. YP_077905, [25]) was 62% and 76%, respectively. CotA of *B. pumilus *consists of 510 amino acids (hypothetical molecular weight: 58.6 kDa), which is close to the length of 513 amino acids for both the *B. subtilis *and *B. licheniformis *laccases. The ligands which bind T1 copper (M502, H419, C492, H497), T2 copper (H105, H422) and T3 copper (H107, H153, H155, H424, H491, H493) in *B. subtilis *CotA [22] were all conserved in *B. pumilus *CotA. The cysteine residues C229 and C322 which have been proposed to form a disulfide bond in *B. subtilis *CotA [29] were also conserved in *B. pumilus *CotA.

### Recombinant expression and purification CotA laccase

For production of *B. pumilus *laccase with *E. coli *JM109 (pBpL6) special growth and induction procedures were applied which had been described previously [26]. Similarly to *B. subtilis *CotA, the use of reduced cultivation temperature before and after induction, the addition of high concentrations of Cu^2+ ^at induction and static incubation in the second phase of CotA expression as stated in materials and methods yielded maximal amounts of fully copper-loaded, active *B. pumilus *enzyme.

**Figure 1:**
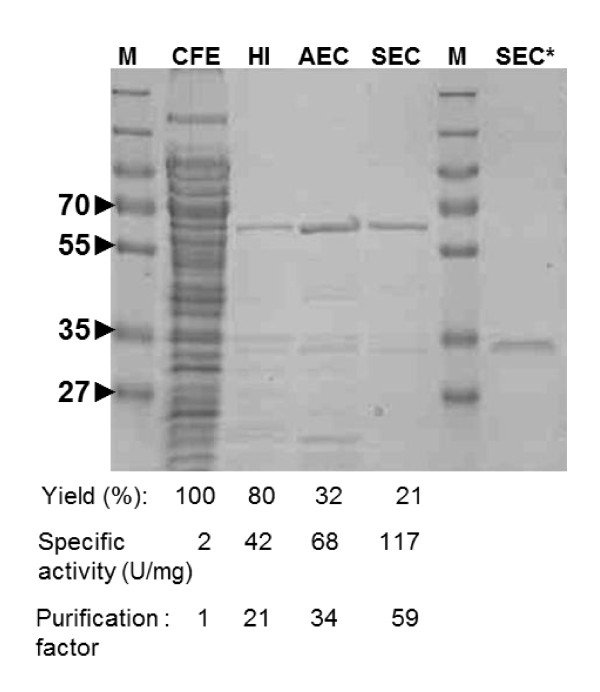
**SDS-PAGE analysis of CotA purification**. Samples from cell free extracts (CFE), heat incubation (HI), anion exchange chromatography (AEC) and size exclusion chromatography (SEC) were denatured for 20 min at 95°C in SDS loading buffer containing β-mercapthoethanol. Sample SEC* was incubated for 5 min at 95° in SDS loading buffer containing β-mercapthoethanol. M, marker. Specific activity was determined using the standard ABTS oxidation assay.

The supernatant from cells expressing laccase was incubated at 70°C for 20 min, as described for other *Bacillus *laccases expressed in *E. coli *[25,30]. This treatment results in denaturation of most *E. coli *proteins and is used to enrich heat resistant proteins such as spore-coat associated laccases. Above 90% of the initial laccase activity was recovered in the supernatant after centrifugation of heat-treated lysate. Subsequent column purifications included anion exchange and size exclusion chromatography, yielding a single protein band of approximately 30 kDa, as shown by SDS-PAGE analysis in Figure 1. The apparent molecular weight of the expressed laccase was much lower than the expected one of approximately 58 kDa, when proteins were separated by SDS-PAGE under standard conditions (5 min, 95°C, in buffer containing 6% SDS and 10% β-mercapthoethanol). However, when the protein was boiled for 20 min, the band migrated at the expected molecular weight, suggesting that full denaturation of the protein required extra heat.

### Biochemical properties of *B. pumilus *laccase CotA

Purified protein samples showed a strong blue color, and copper content determination revealed a molar copper to protein ratio of 3.9, affirming a fully copper-loaded enzyme. The pH optima of CotA activity were determined to be around pH 4 for ABTS, pH 7 for 2,6-DMP and pH 6.5 for SGZ. These values were very similar to those found for other *Bacillus *CotA laccases [25,28]. The temperature optimum for ABTS oxidation was determined to be between 55-75°C. For 2,6-DMP oxidation a steady increase of activity up to 70°C was monitored, again demonstrating the high temperature tolerance of CotA laccase. For detailed temperature-activity and pH-activity profiles see Additional file 2.

**Figure 2:**
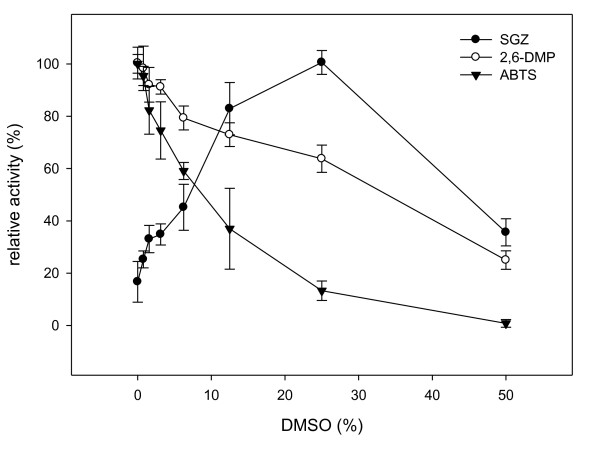
**Effect of DMSO upon CotA activity from *B. pumilus***. Effect of DMSO present in the reaction mixture upon initial rate of SGZ (•), ABTS (▼) and 2,6-DMP (○) oxidation. Relative activity was normalized to the maximal activity achieved for each substrate. All data points represent mean values from triplicate determinations.

Kinetic parameters of recombinant *B. pumilus *laccase for ABTS, 2,6-DMP and SGZ oxidation were determined and are shown in Table 1. Michaelis-Menten plots for all substrates are given in Additional file 3. The values obtained for ABTS and 2,6-DMP were more similar to those obtained for *B. subtilis *than for *B. licheniformis *laccase [25,26]. It was not possible to determine the kinetic parameters for SGZ due to its low solubility. The observed kinetics did not fit a Michaelis-Menten equation. Instead, only a linear correlation was observed. This was true either when low SGZ (6-78 μM) and DMSO concentrations (< 0.8% v/v) or high SGZ (0.08-1.25 mM) and DMSO (25% v/v, leading to maximal rates of SGZ oxidation, see Fig. 2) concentrations were used. Hence, no *K*_M _can be given for SGZ oxidation. Nevertheless, a rough estimate of *k*_cat _was deduced from the highest observed reaction rates measured with either < 0.8% (v/v) or 25% (v/v) DMSO (data marked with * in Table 1).

**Table 1:** Kinetic constants of purified laccase from *Bacillus *species for ABTS, 2,6-DMP and SGZ oxidation.

Substrate									
**Laccases**	**ABTS**			**2, 6-DMP**			**SGZ**		
	
	***K*_M _(μM)**	***k*_cat _(s^-1^)**	**Assay conditions**	***K*_M _(μM)**	***k*_cat _(s^-1^)**	**Assay conditions**	***K*_M _(μM)**	***k*_cat _(s^-1^)**	**Assay conditions**

*B. pumilus*	80 ± 4	291 ± 2.7	0.6 μM-10 mM, pH 4, RT	680 ± 27	11 ± 0.1	0.6 μM-10 mM, pH 7, RT	n.d.	*4.9	6 - 78 μM, <0.8% (v/v) DMSO pH 6.5, RT
							
							n.d.	*66 ± 1.5	0.04 μM-1.25 mM (25% (v/v) DMSO), pH 7, RT

*B. subtilis *[26]	124 ± 17	322 ± 20	10 -200 μM pH 4, 37°C	216 ± 35	29 ± 4	10 -200 μM, pH 7, 37°C	18 ± 3	80 ± 4	1 -100 μM, pH 7, 37°C

*B. licheniformis *[25]	6.5 ± 0.2	83	5 -500 μM pH 4, RT	56.7 ± 1	28	5 -1000 μM, pH 7, RT	4.3 ± 0.2	100	1.25 -50 μM, pH 7, RT

Substrate ranges covered, pH and temperature are listed. n.m.: not measurable, due to substrate solubility. * The value was deduced from the highest observed rate.

SGZ required the presence of DMSO to enable its solubility. The effect of increasing concentrations of DMSO upon laccase activity was therefore investigated. To this end, ABTS, 2,6-DMP and SGZ oxidations were measured in the presence of increasing DMSO concentrations (Figure 2). It was found that SGZ oxidation rates were enhanced with increasing concentrations of DMSO with an optimum at around 25% (v/v). Concentrations above 50% (up to 90%) did not result in further enhanced activity, but rather deactivated the enzyme rapidly (data not shown). For ABTS and 2,6-DMP any addition of DMSO led to a gradually decreased oxidation rate; however, the effect was much more severe for ABTS.

The kinetic stability upon storage of the enzyme was also investigated. Protein samples were incubated in McIlvaine buffer at pH 5 and 7, potassium phosphate buffer at pH 7 and ddH_2_O at 4, 25, 45 and 65°C. ABTS oxidation was measured periodically. The half-life of laccase is summarized in Table 2. Within 1 hour, all laccase preparations in McIlvaine buffer pH 5 showed over 75% loss of activity. Enzyme samples stored in either McIlvaine or phosphate buffer at pH 7 showed over 50% loss in activity after 1 h. Unexpectedly, this significant loss was not detected for the samples stored in deionized water.

**Table 2:** Half-life of laccase measured by ABTS oxidation.

	4°C	25°C	45°C	65°C
McIllvaine buffer pH 7	1.0	0.75	0.32	0.28

Potassium phosphate buffer pH 7	0.93	0.79	0.45	0.34

Deionized water	144	40.0	19.3	3.5

The purified protein was stored in different solvents and at various temperatures. Half-lives are given in hours.

### Substrate range of *B. pumilus *laccase CotA

The catalytic activity of purified CotA was screened against a panel of 37 structurally different potential substrates of both synthetic and natural origin. The activity was measured at a substrate concentration of 1 mM. A UV-Vis scanning between 230-700 nm was recorded prior to laccase addition and after 48 h reaction time. By this method only substrates leading to a changed UV spectrum upon oxidation were detectable. From the 37 compounds tested, a change of absorbance was detected for 18 compounds (Table 3). Control reactions lacking laccase were performed in parallel, to exclude the possibility of non-enzymatic oxidation.

**Table 3:** Substrate screen for laccase activity and IC decolorization Change of absorbance (Δ*A*): + (change of absorbance), - (no change of absorbance).

Structure	Compound	n°	∆*A*	IC
	R1 = R2 = H	1	-	0
	
	R1 = H, R2 = OH	2	-	0
	
	R1 = OH, R2 = H	3	-	0
	
	R1 = R2 = OH	4	+	0
	
	R1 = OH, R2 = OCH_3_	5	+	25

	R1 = OH, R2 = R3 = H	6	-	0
	
	R1 = R2 = OH, R3 = H	7	+	0
	
	R1 = R2 = R3= OH	8	+	0
	
	R1 = OH, R2 = R3= OCH_3_	9	+	0
	
	R1 = OH, R2 = NH_2_, R3 = H	10	+	0
	
	R1 = NH_2_, R2 = OH, R3 = H	11	+	0
	
	R1 = OH, R2 = F, R3 = H	12	-	0

	R1 = OH, R2 = H	13	-	0
	
	R1 = OH, R2 = OCH_3_	14	+	40
	
	R1 = OCH_3_, R2 = OH	15	+	0

		16	-	0

	R = H	17	-	0
	
	R = OCH_3_	18	+	>99

	R1 = OH, R2 = R3 = H	19	-	0
	
	R1 = H, R2 = OH, R3 = OCH_3_	20	+	>99

		21	+	0

		22	-	0

	R = H	23	-	0
	
	R = OH	24	+	0

	R = H	25	-	0
	
	R = OH	26	-	0

	R1 = R2 = R3 = H	27	-	0
	
	R1 = R3 = H, R2 = CH_3_	28	-	0
	
	R1 = R3 = CH_3_, R2 = H	29	-	0
	
	R1 = OH, R2 = R3 = H	30	+	0
	
	R1 = OH, R2 = CH_3_, R3 = H	31	+	0
	
	R1 = R3 = OH, R2 = H	32	+	0
	
	R1 = R3 = H, R2 = β-O-Gluc	33	-	0

		34	+	0

		35	-	0

		36	-	0

		37	+	>99

Indigocarmine decolorization (%) (IC) is given compared to the control reaction lacking laccase.

In addition, all substrates were tested for their ability to function as mediator in the decolorization of the dye IC (Table 3). The relative decrease of absorbance was determined spectrophotometrically as a measure of the degree of mediated oxidation of IC. For the mediator screen, 1 mM IC was combined with 0.1 mM of the substrate. The results are summarized in Table 3.

Amongst the 37 compounds tested, 18 showed a change of absorbance as a result of laccase promoted oxidation. In turn, three of these compounds, ABTS (**37**), ACS (**18**) and syringaldehyde (**20**) functioned as mediators and completely decolorized IC within 3 hours. Within 48 hours, two compounds promoted decolorization of 25% (**5) **and 40% (**14**), respectively. Laccase alone lead to a decolorization of 20% within 48 hours at pH 6.8. All other compounds did not lead to a decolorization of more than 20% and therefore cannot be considered as mediators for IC oxidation.

**Figure 3:**
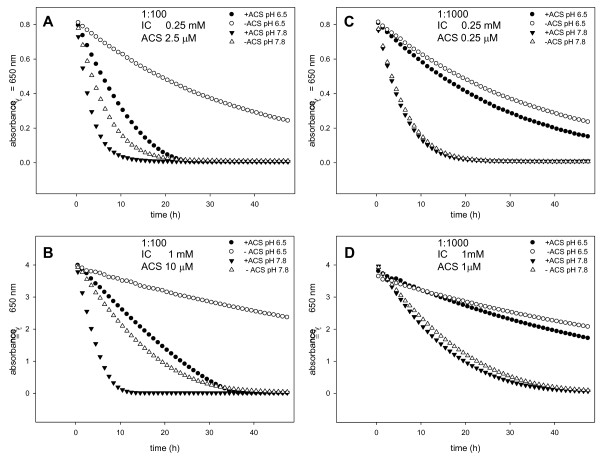
**IC transformation**. Time course of IC degradation by purified laccase from *B. pumilus *monitored spectroscopically at λ = 650 nm over a total of 48 h at 37°C. Reactions were conducted at pH 6.5 and 7.8, in the presence or absence of ACS. (A) 0.25 mM IC and ACS added at a ratio of 1:100. (B) 1 mM IC and ACS added at a ratio 1:100. (C) 0.25 mM IC and ACS added at a ratio of 1:1000. (D) 1 mM IC and ACS added at a ratio of 1:1000.

ACS was selected for further studies and its potential as a mediator for the decolorization of IC was evaluated. In the screening, experiments were conducted with 0.1 mM ACS and 1 mM IC (1:10 ratio). This ratio was decreased to 1:100 (Figure 3A and 3B) and 1:1000 (Figure 3C and 3D) with IC starting concentrations of 0.25 mM and 1 mM, respectively. Furthermore, the pH effect was evaluated by performing the reactions at pH 6.5 and 7.8. Direct decolorization of IC by laccase was monitored in reactions lacking ACS.

Both mediated and non-mediated reactions were more efficient at higher pH. Although IC was partially (at pH 6.5) and completely (at pH 7.8) decolorized by laccase alone within 48 h, the mediated reactions proceeded faster, most notably at IC:ACS ratio of 1:100. It was found that mediated reactions at IC:ACS ratio of 1:100 completely decolorized IC at both pH values. However, at IC:ACS ratio of 1:1000, only reactions at pH 7.8 were completed. Independently of the pH, the rates of ACS mediated reactions performed at an IC:ACS ratio of 1:1000 and non-mediated reactions were almost the same. This indicated that ACS concentrations significantly below the *K*_m_, which was determined to be 30 mM, had no influence on the reaction kinetics.

Decolorization reactions were also conducted with 10 mM IC and 1:100 and 1:1000 ratios of IC:ACS at pH 6.5 and 7.8. As a control, direct oxidation was monitored in reactions lacking ACS. Although the initial absorbance could not be detected spectrophotometrically (OD_650 nm _> 4), complete decolorization within 72 hours was observed for the reaction performed at an IC:ACS ratio of 1:100 and pH 7.8. No measurable bleaching was observed for the non-mediated reaction or any of the other control reactions conducted. In reactions which lead to complete decolorization of IC at λ = 650 nm, a red soluble reaction product with an absorbance maximum at λ = 550 nm was formed. The formation of a red product during laccase-catalyzed IC transformation has previously been reported and presumably is due to the condensation of two IC molecules [31,32].

## Discussion

Laccases are versatile biocatalysts for industrial applications. Much research has been done with fungal laccases; however, recently more information has become available also for bacterial laccases [33]. We have identified a novel CotA-type laccase by homology searches in public protein databases. The laccase from *Bacillus pumilus *DSM 27 was cloned, purified and biochemically characterized. The purified fully copper loaded protein was found to be a monomeric 58 kDa recombinant protein, which exhibited a blue color, due to the characteristic absorbance of the chelated T1 copper site at 600 nm [2].

Laccase from *B. pumilus *constitutes a highly thermostable protein. The pH optimum for ABTS, 2,6-DMP and SGZ, as well as the temperature optimum between 55-75°C were similar to *B. subtilis *and *B. licheniformis *laccases [25,26]. Thermal stability studies showed that our laccase maintained activity best when stored in water. Therefore, selection of the appropriate storage solvent seems to be crucial for *Bacillus *laccases in order to maintain high levels of active protein for either storage or in biotransformation reactions.

Kinetic constants for ABTS and 2,6-DMP were within the range of data reported for *B. subtilis *and compared favorably to that of fungal laccases [34,35]. However, the *K*_M _for SGZ was not measurable and a *k*_cat _value of 66 s^-1 ^could only be estimated from the highest observed reaction rate at 1.25 mM SGZ and 25% (v/v) DMSO. The initial rate of SGZ oxidation was highest when the reaction mixture was supplemented with 25% (v/v) DMSO. By contrast, any addition of DMSO to reactions performed with ABTS and 2,6-DMP lead to an immediate decline of activity, probably due partial denaturation or inhibition of the enzyme. In the case of SGZ, the increased solubility of the substrate at increasing DMSO concentrations presumably over-compensates negative effects of the co-solvent. An inhibitory effect of DMSO on laccase catalyzed oxidation of 2,6-DMP has been previously reported for an enzyme from *Pyricularia oryzae *[36]. Increasing amounts of DMSO were also added to laccase from *Trametes *sp. and the conversion of 4-hydroxybiphenyl decreased with increasing DMSO concentrations. However, the study was only carried out for DMSO concentrations between 30 to 70%, as the substrate was not soluble below 30% [37]. We conclude from our data that DMSO up to 25% (v/v) can be used as a co-solvent for enhancing the solubility of hydrophobic substrates in biotransformations with CotA-type laccases.

To explore the potential of laccase as a biocatalyst a substrate screen for 37 non-comprehensive compounds was performed. Both, different substituted phenols and non-phenolic substrates were tested qualitatively. It was found that purified laccase from *B. pumilus *exhibited a broad substrate spectrum. Oxidizing activity towards 18 compounds was detected, which was in many cases accompanied by the associated color formation. No activity was detected for the non-phenolic substrates **1**, **22**, **25**, **26**, **35 **and **36**, which might be due to the lack of a changed spectroscopic signal upon oxidation. However, it was observed that phenolic substrates were preferably oxidized when at least one -OH or -OCH_3 _with a lone pair of electrons was adjacent to the phenol, such as substrates **4**, **5**, **7**, **8**, **9**, **10**, **11**, **14**, **15**, **18**, **20**, **21**, **24**, **30**, **31**, **32**, and **34**. For substrates **16**, **17 **and **19**, no activity could be detected, although one *ortho *position was substituted with an -OCH_3_. For these compounds to function as substrates, a substitution at both *ortho *positions might be vital. Kinetic studies with a number of basidomycetes suggested that the higher activity for sinapic acid compared to ferulic acid could be the result of the additional -OCH_3 _group [38]. A similar correlation between activity and substitution pattern has been described for laccase from *Trametes trogii*, *Streptomyces ipomoea, Pleurotus ostreatus *and *B. licheniformis *[25,39-41]. The fact that the *ortho *-H or -CH_3 _substituted substrates **2**, **3**, **6**, **13**, **23**, **27, 28**, **29 **and **33 **were not oxidized is in line with the proposed requirement for the aforementioned substitution pattern. The oxidizing activity of laccase might be rationalized based on the principle of inductive and mesomeric effects. The substrate screen revealed that the enzyme preferably oxidizes substituted phenols with at least one *ortho *- or *para *- substituent bearing a lone pair of electrons, contributing to the positive mesomeric effect. As a consequence, the electron density at the phenoxy-OH is increased, thus facilitating an electron abstraction that yields a phenoxy radical. Compound **12 **is not oxidized because the negative inductive effect of halogens such as fluorine is dominating over the positive mesomeric effect.

The substrates were also tested for their potential to act as mediator in the IC decolorization. For three compounds, namely ABTS (**31**), syringaldehyde (**14**) and ACS (**12**), a complete decolorization was achieved using LMS with *B. pumilus *laccase. A number of groups have reported on the direct or mediated IC decolorization using bacterial as well as fungal laccase [7,14,15,17,19,42]. Routinely, reactions were carried out using 20-100 μM IC in combination with various mediators that were added in equimolar amounts or in a molar ratio of 1:10. Successfully employed mediators, which led to best decolorization results, were syringaldehyde, acetosyringone and violuric acid. A true mediator should be added in catalytic amounts. Yet, in most studies on dye decolorization the mediators are added in high stoichiometric amounts. In our study, we reduced the mediator to dye ratio to 1:100 and found that ACS functioned as a "true" mediator, which significantly enhanced the decolorization rate. Only three of the 18 substrates tested functioned as mediators for the decolorization of IC. The other identified primary substrates might still function as mediators for the oxidation of other dyes/secondary substrates.

## Conclusions

In summary, laccase from *B. pumilus *was cloned, expressed and biochemically characterized. It was shown to constitute a versatile biocatalyst and was successfully applied in the decolorization of up to 10 mM IC in the presence of catalytic amounts of mediators. We concluded that CotA from *B. pumilus *has great potential as biocatalyst due to its thermo stability, pH optimum in the neutral to alkaline range and broad substrate spectrum.

## List of abbreviations

2,2': Azino-bis(3-ethylbenzthiazoline-6-sulphonic acid) (ABTS);

2,6: Dimethoxyphenol (2,6-DMP);

(SGZ): Syringaldazine;

(LMS): Laccase mediator systems;

(IC): Indigocarmine;

## Competing interests

The authors declare that they have no competing interests.

## Authors' contributions

RR participated in the design of the study, carried out the experiment and in writing the manuscript. JI participated in the design of the study, carried out the cloning and helped to draft the manuscript. LMT conceived the study, provided financial and administrative support and participated in the design of the study and writing the manuscript. All authors read and approved the final manuscript.

## Supplementary Material

Additional file 1 Map of the constructed plasmid pBpL6 used for expression of CotA laccase from *B. pumilus* Click here for file

Additional file 2 **pH dependence and temperature optimum of CotA activity from *B. pumilus***. (A) Oxidation of ABTS (■ 0.5 mM), 2,6-DMP (• 0.3 mM) and SGZ (▲ 0.05 mM) by the purified laccase as a function of pH at 25°C. Relative activities were normalized to the maximal activity achieved for each substrate at the optimum pH, which was taken as 100% (corresponds to 200, 4.3 and 5.4 U/mg for ABTS, 2,6-DMP and SGZ respectively). The oxidation by laccase as a function of temperature is given relative to the highest activity recorded, which was taken as 100%. This corresponds to 24.8 U/mg for (B) 2,6-DMP (0.3 mM, pH 7) and 857 U/mg for (C) ABTS (0.5 mM, pH 4). All data points represent mean values from triplicate determinations. Click here for file

Additional file 3 **Michaelis-Menten plots obtained with purified CotA laccase from *B. pumilus***. The specific activity (U/mg) was plotted *versus *the substrate concentration for (A) ABTS, (B) 2,6-DMP and (C) SGZ (25% (v/v) DMSO). All data points represent mean values ± standard error from triplicate determinations. Click here for file
