# Supplementary material for: Bacillus pumilus laccase: a heat stable enzyme with a wide substrate spectrum
Source: BMC Biotechnol. 2011 Jan 25;11:9. doi: 10.1186/1472-6750-11-9 (PMC3041658; doi:10.1186/1472-6750-11-9)
Supplement: Additional file 1 — Map of the constructed plasmid pBpL6 used for expression of CotA laccase from B. pumilus [file 1472-6750-11-9-S1.DOC]

**pBpL6**

*Hin*dIII

*Xho*I
