# Supplementary figures and images for: Bacillus pumilus laccase: a heat stable enzyme with a wide substrate spectrum
Source: BMC Biotechnol. 2011 Jan 25;11:9. doi: 10.1186/1472-6750-11-9 (PMC3041658; doi:10.1186/1472-6750-11-9)

## Slide 1
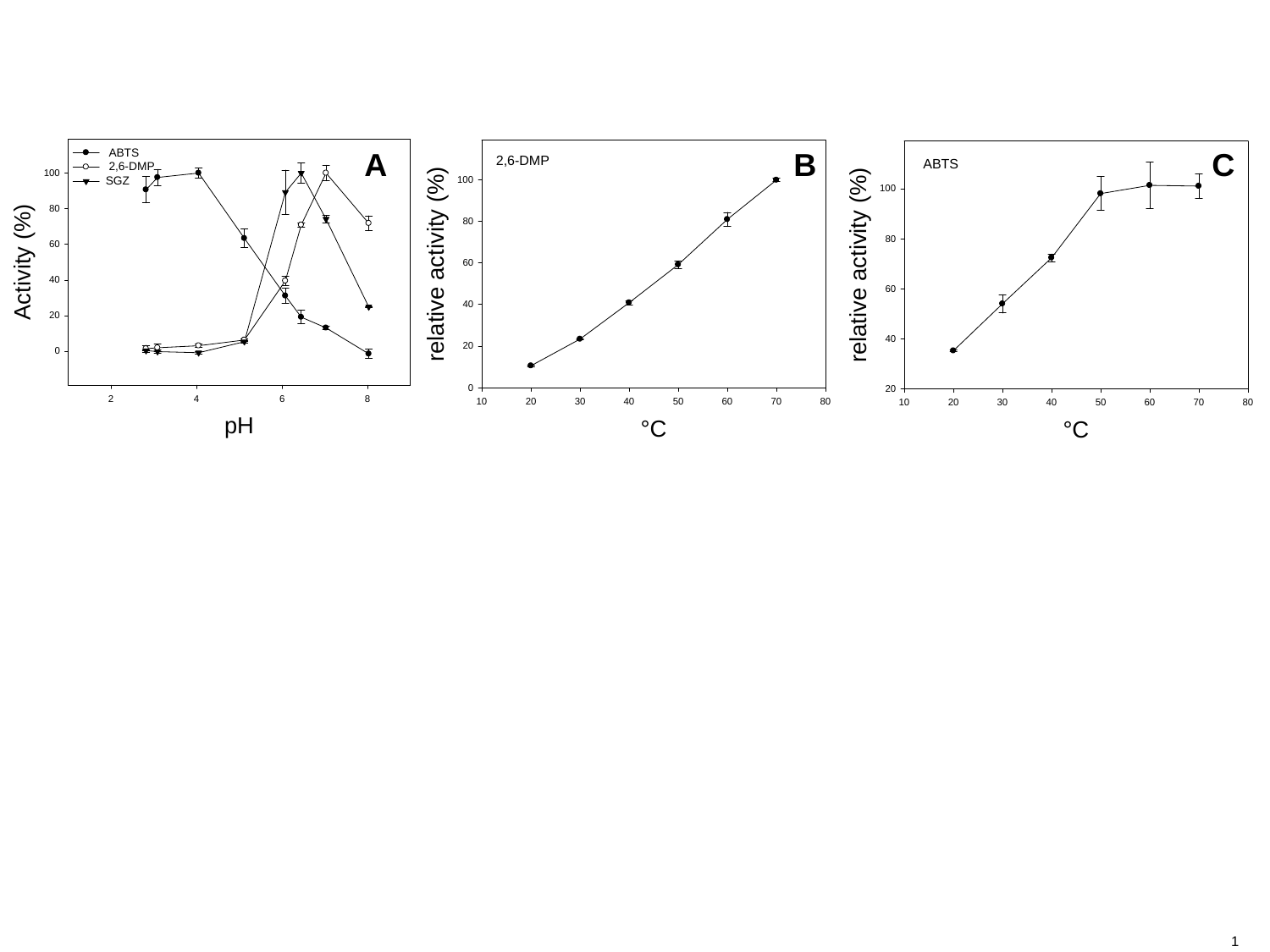

A B		 C
<number>

Supplement: Additional file 2 — pH dependence and temperature optimum of CotA activity from B. pumilus. (A) Oxidation of ABTS (■ 0.5 mM), 2,6-DMP (• 0.3 mM) and SGZ (▲ 0.05 mM) by the purified laccase as a function of pH at 25°C. Relative activities were normalized to the maximal activity achieved for each substrate at the optimum pH, which was taken as 100% (corresponds to 200, 4.3 and 5.4 U/mg for ABTS, 2,6-DMP and SGZ respectively). The oxidation by laccase as a function of temperature is given relative to the highest activity recorded, which was taken as 100%. This corresponds to 24.8 U/mg for (B) 2,6-DMP (0.3 mM, pH 7) and 857 U/mg for (C) ABTS (0.5 mM, pH 4). All data points represent mean values from triplicate determinations. [file 1472-6750-11-9-S2.PPT]

## Slide 1
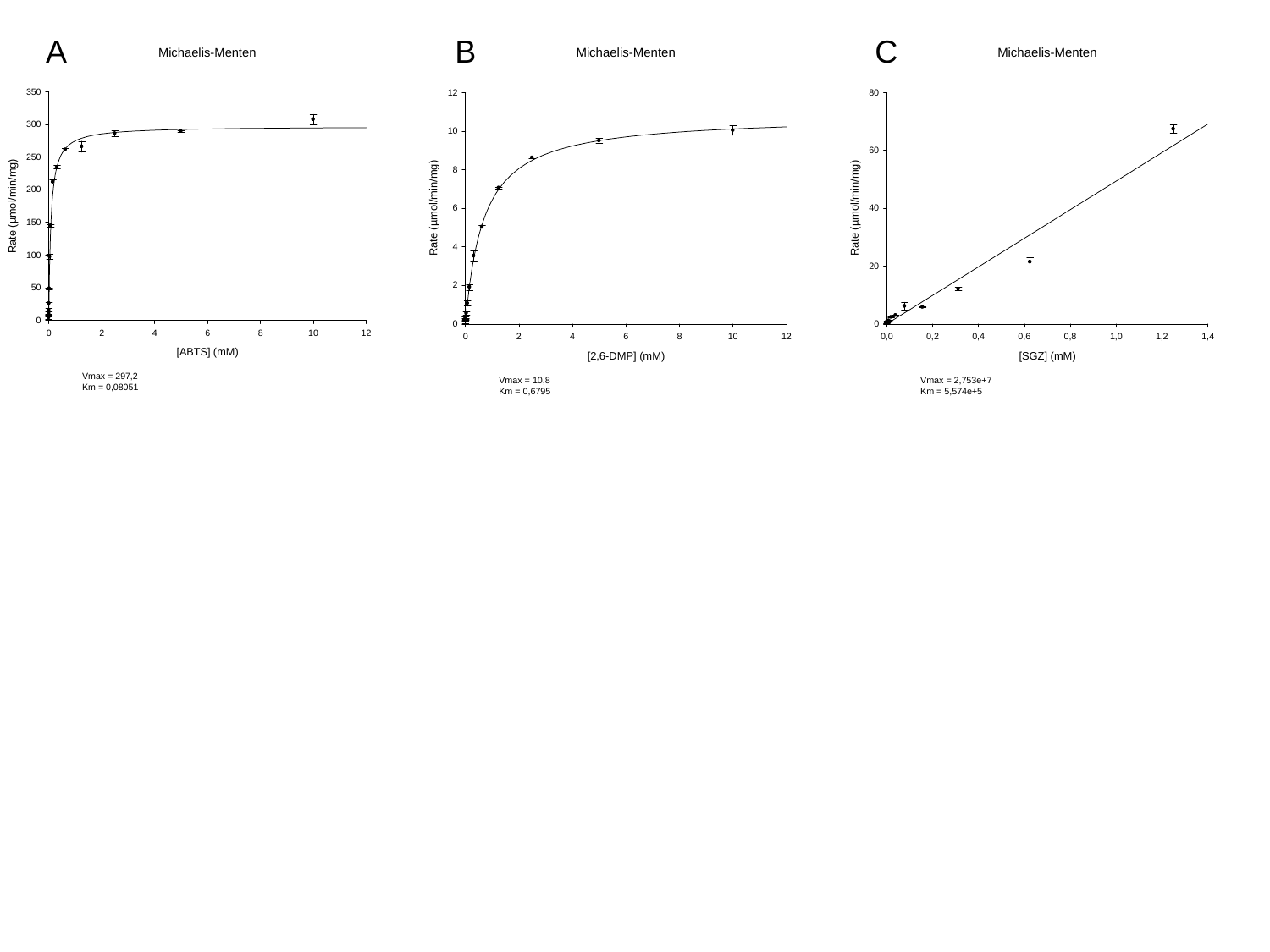

A B C

Supplement: Additional file 3 — Michaelis-Menten plots obtained with purified CotA laccase from B. pumilus. The specific activity (U/mg) was plotted versus the substrate concentration for (A) ABTS, (B) 2,6-DMP and (C) SGZ (25% (v/v) DMSO). All data points represent mean values ± standard error from triplicate determinations. [file 1472-6750-11-9-S3.PPT]
